# Severe Recurrent Fever Episodes With Clinical Diagnosis of Hemophagocytic Lymphohistiocytosis, Incomplete Kawasaki Disease and Systemic-Onset Juvenile Idiopathic Arthritis: A Case Report and Literature Review

**DOI:** 10.3389/fped.2020.00093

**Published:** 2020-03-10

**Authors:** Hongkun Jiang, Zhiliang Yang

**Affiliations:** Department of Pediatrics, The First Hospital of China Medical University, Shenyang, China

**Keywords:** incomplete Kawasaki disease, hemophagocytic lymphohistiocytosis, systemic-onset juvenile idiopathic arthritis, recurrent fever, PFAPA

## Abstract

The pathogeneses of recurrent fever are quite complicated when excluding repeated infections. Recurrent fever is a common symptom for autoinflammatory diseases, relapse of Systemic-onset juvenile idiopathic arthritis (SoJIA) and recurrent Kawasaki disease (KD). There are no specific diagnostic laboratory tests for the diseases. Some studies showed that KD was the precursor of hemophagocytic lymphohistiocytosis (HLH). Macrophage activation syndrome (MAS) is another form of HLH in SoJIA. Cytokine disturbances are considered to be involved in the pathogenesis of the diseases. We describe a Chinese female toddler that developed three separate fever episodes with eventual diagnose of SoJIA within about 10 months. The first episode was diagnosed as IKD, immunoglobulin nonresponsive KD, and HLH. The second and third episodes were diagnosed as IKD and SoJIA, respectively. The fever was hard to be relieved by antipyretics, and the peak axillary temperature was above 40°C. For every fever episode, infections were excluded. For the first episode, trends over time of hemoglobin, platelets, fibrinogen, and triglycerides indicated HLH, which was finally diagnosed and treated according to the HLH-2004 protocol. For the second episode 6 months later, after excluding an HLH relapse and infections, IKD was finally diagnosed. Oral aspirin was administered, and the HLH treatment was ceased. The third episode occurred 3 months later, and SoJIA was finally diagnosed. For each episode, except for relative tests, we only tested for cytokines interleukin-1β, interleukin-6, and interferon-γ, due to limited laboratory test availability. These cytokines were elevated during remission and rose much higher in the fever phases. The case showed the difficulty to differentiating the recurrent fever in clinical practice. Surveillance of routine laboratory parameters over time might reveal a trend that indicates possible disease, even when parameter values do not meet diagnostic criteria. Changes in cytokine profiles are promising markers for differentiating recurrent fever diseases in future. An unknown immunological defect for the case may contribute to the recurrent immunological insults, and we are following up the recurrence of fever episode.

## Introduction

A fever of unknown origin (FUO) was considered as an unexplained fever that persisted longer than 1 week in a child with negative findings in preliminary investigations (1). Recurrent fevers also are common in children. Excluding the repeated infections, the differentiating diagnosis for the fevers is a major topic. Recurrent fevers can occur in autoinflammatory diseases (2), recurrent Kawasaki disease (KD) (3, 4) and relapse of Systemic-onset juvenile idiopathic arthritis (SoJIA) (5). Autoinflammatory diseases include Periodic Fever, Aphthous Stomatitis, Pharyngitis, and Adenitis (PFAPA) syndrome, Familial Mediterranean fever (FMF), cryopyrin-associated periodic syndrome (CAPS), tumor necrosis factor receptor-associated periodic fever syndrome (TRAPS), and hyperimmunoglobulinemia D and periodic fever syndrome/mevalonate kinase deficiency (HIDS/MVKD), which are characterized by periodic fever (2, 6). SoJIA is usually considered in patients with fever of ≥2 weeks and arthritis in ≥1 joint with some other features after excluding some diseases (7). But some patients may not present with any arthritis initially, and this can quite torturous for clinicians to make the diagnosis of SoJIA.

Macrophage activation syndrome (MAS) is considered to be another form of hemophagocytic lymphohistiocytosis (HLH) associated with connective tissue diseases (CTD), particularly SoJIA (8). MAS usually observed after the diagnoses of CTD, and HLH is the usually diagnosis for patients without diagnoses of CTD. KD was considered to be a precursor of HLH (9–11), and the incidence of SoJIA in HLH or KD patients has not been reported. HLH/MAS is only reported in one case from 114 cases of HIDS/MVKD (12) and not reported in FMF, CAPS, TRAPS and PFAPA. All the diseases were associated with cytokines contributes. Various cytokines can induce different diseases that commonly manifest as fever. Interleukin-6 (IL-6) is associated with Kawasaki disease (KD), particularly incomplete KD (IKD); and IL-1 and IL-6 also play critical roles in the pathogenesis of SoJIA (13). PFAPA syndrome is also characterized by a cytokine dysfunction (14, 15), with elevated TNF-α, IL-1β, and IL-6 (16). These observations might comprise the basis for the overlapping manifestations of KD, HLH, SoJIA, and PFAPA.

The diagnostic criteria for these diseases were mostly established according to the characteristics of classic cases at classic phases. For atypical cases or cases at an early stage, the diagnosis can be quite difficult and delayed. Here, we describe a Chinese toddler who developed three separate fever episodes within about 10 months. The first episode was diagnosed as IKD, immunoglobulin (IVIG) nonresponsive KD, and HLH. The second episode was diagnosed as IKD. The third episode was diagnosed as SoJIA according to clinical and laboratory findings. No previous study has reported such a case manifested like PAFPA but also meet the diagnosis of IKD and HLH; thus, this report highlights the difficulty for diagnosis in atypical recurrent FUO cases.

## Case-Report

### The First Fever Episode

A 16-month-old Chinese girl was admitted with a chief complaint of 7 days fever without obvious cause. Her medical and family histories were unremarkable. Her parents had no consanguinity. The peak axillary temperature was 40.2°C. The fever subsided for about 6 h by oral ibuprofen, but then it reoccurred. She displayed no coughing or wheezing. Oral erythromycin and esberitox were administered for 5 days at an outpatient clinic. Three days before admission, rashes occurred in the face and extremities without itching. Upon physical examination, the rash had spread all over the whole body, but it faded on pressure and displayed no desquamation. Several enlarged anterior cervical lymph nodes were found. There were no other obvious signs. Laboratory tests were performed to evaluate blood samples. The white blood cell count (WBC) was 7.46 × 10^9^/L (normal range [NR]: 4–10 × 10^9^/L), with 32.5% neutrophils; the hemoglobin (HGB) was 104 g/L (NR: 110–140 g/L); the platelet count (PLT) was 160 × 10^9^/L (NR: 100–300 × 10^9^/L); the C-reactive protein (CRP) level was 24.9 mg/L (NR: 0–5 mg/L); the procalcitonin (PCT) level was 1.18 ng/L (NR: 0–0.05 ng/L); the ferritin level was >2,000 μg/L (NR: 13–150 μg/L); and the erythrocyte sedimentation rate (ESR) was 7 mm/h (NR: 0–15 mm/h). Immunoglobin M Tests were negative for *Mycoplasma pneumoniae* and Epstein-Barr virus. Sepsis was considered, and intravenous ceftriaxone was prescribed. After 5 days, the fever persisted, and the rash became more obvious when temperature was over 38.5°C. A re-evaluation revealed that CRP increased to 46.5 mg/L and ESR to 52 mm/h, alanine aminotransferase (ALT) was elevated to 631 U/L (NR: 9–40 U/L). IKD was diagnosed and the basis for diagnosis were showed in Table 1 (B). A single dose of IVIG (2 g/kg) was administered in 24 h. However, the fever persisted. Therefore, IVIG-nonresponsive KD was considered. SoJIA was also considered but not diagnosed because the case did not meet the criteria of ILAR (17) and she showed rapid deterioration in laboratory tests. During this period, the laboratory tests revealed that HGB, PLT, and fibrinogen levels had progressively decreased: HGB declined from 99 to 88 g/L, PLT declined from 172 to 108 × 10^9^/L, and fibrinogen declined from 2.6 to 1.31 g/L (NR: 2.0–4.0 g/L). In contrast, the triglycerides increased to 5.73 mmol/L (NR: 0–1.7 mmol/L) and ferritin remained constant at >2,000 μg/L. Upon a physical examination, the liver was enlarged: its lower edge had extended 4 cm below the costal margin, but no splenomegaly was found. HLH was highly suspected, because four out of the eight criteria were met, according to the HLH2004 criteria (18). Further evaluations were performed for ascertaining HLH. Because the HLH diagnosis could not be readily confirmed, the parents provided written informed consent to administer dexamethasone and cyclosporine A (CSA). The fever subsided within 1 day after initiating this treatment, and the rashes disappeared after 2 days. The HLH test results were received after 5 days. They showed that soluble CD25 (sCD25) was 43,473 pg/ml (NR: <6,400 pg/mL), and CD107a had increased to 5.17% (NR: >10%) after stimulating natural killer cells. These results indicated that natural killer cell activity was defective. Hemophagocytosis was not detected in bone marrow aspirates. Antibody detection was negative for antibodies against Rheumatoid Factor (RF), ANA, ds-DNA, Sm, SS-A, SS-B, U1RNP, RO-52, SCL-70, PM-Scl, and ENA-Jo-1. No mutation was detected in the genes associated with primary HLH, including *PRF1, UNC13D, STX11, STXBP2, LYST, RAB27A, AP3B1, SH2D1A, XIAP*, and *ITK*. Secondary HLH was diagnosed according to fulfilling five of the eight criteria for HLH and negative genetic detection for primary HLH, and etoposide was administered according to the HLH-2004 protocol. Liver-protective therapy [intravenous glutathione (0.6 g/day) and vitamin C (100 mg/Kg.day)] was performed simultaneously. The treatment was administered without complications, and the patient improved without recurrence of the fever or rash. In the next 6 months, the patient was in generally good condition with the treatment regimen.

**Table 1:** Diagnosis evaluation for suspected IKD from literatures and clinical diagnostic basis for our case.

**A: Diagnosis clinical criteria for clinical KD**		**B: Diagnostic basis for IKD for the first fever episode**	**C: Diagnostic basis for IKD for the second fever episode**
Essential criteria	Fever ≥5 days	YES	YES
At least four of five principal clinical features	Erythema and cracking of lips, strawberry tongue, and/or erythema of oral and pharyngeal mucosa		
	Bilateral bulbar conjunctival injection without exudate		
	Rash: maculopapular, diffuse erythroderma, or erythema multiforme-like	YES	YES
	Erythema and edema of the hands and feet in acute phase and/or periungual desquamation in subacute phase		
	Cervical lymphadenopathy (≥1.5 cm diameter), usually unilateral	YES	YES
Evaluation for suspected IKD: CRP≥3.0 mg/dL and/or ESR≥ 40 mm/h		YES	YES
At least three of the laboratory findings	Anemia for age	YES	YES
	Platelets ≥ 450 × 10^3^/μL (450 × 10^9^/L) after first week	YES	YES
	Hypoalbuminemia ≤ 3.0 g/dL (30 g/L)		YES
	Elevated liver enzyme levels	YES	
	White blood cell count ≥ 15,000/μL (15.0 × 10^9^/L)		
	Urine ≥ 10 WBC/hpf		
OR	Positive echocardiography	NO	NO

*A: The diagnosis clinical criteria for clinical KD and evaluation for suspected IKD from lecture 28 and 29*.

### The Second Fever Episode

She was admitted after a fever that lasted 1 day. The patient had a slightly runny nose with no cough or pharyngalgia. At the time of admission, she took oral dexamethasone (5 mg) and CSA (25 mg) every day for HLH. The fever was only slightly relieved with oral ibuprophen or acetaminophen. The peak axillary temperature was 40.3°C. The throat was slightly red, and enlarged cervical lymph nodes were found, and the remainder of physical examination was unremarkable. Laboratory tests revealed that the WBC was 11.56 × 10^9^/L (NR: 4–15 × 10^9^/L), HGB was 116 g/L (NR: 110–180 g/L), CRP was 193.9 mg/L (NR: 0–5 mg/L), and PCT was 2.87 ng/L (NR: 0–0.05 ng/L). Sepsis was considered initially, and ceftriaxone was prescribed. Due to the immunocompromised condition, IVIG (400 mg/kg/d) was also administered to control infections. Three days later, the fever persisted. The re-evaluation revealed that the WBC was 6.36 × 10^9^/L, HGB was 95 g/L, CRP was 105.1 mg/L, PCT was 3.36 mg/L, and ESR was 72 mm/h. The evaluation for diagnosing HLH relapse revealed that the ferritin, triglycerides, fibrinogen, and sCD25 were within normal ranges. The antibiotic was changed to meropenem, and IVIG was continued for 5 days. Two days later, the WBC was 8.73 × 10^9^/L, HGB was 88 g/L, CRP was 63.7 mg/L, PCT was 0.96 ng/mL, ESR was 83 mm/h, ferritin was 175.9 μg/L, and albumin was 29.3 g/L. The fever persisted during the daytime, but settled down suddenly at night. At the same time, skin rashes appeared on the trunk. We noticed that the HCG had declined from 116 to 88 g/L. The blood culture for bacteria was negative. After 5 days of IVIG (the total dose was 2 g/kg), the fever ceased, but the CRP and PCT remained markedly elevated. Consequently, IKD was highly suspected. Coronary artery ectasia (CAE) was not found with echocardiography. Aspirin (30 mg/kg/d) was administered, and meropenem was stopped after 3 days. The skin rashes disappeared the next day. Three days later, the HGB was 96 g/L and the PLT had increased to 686 × 10^9^/L. The fever did not relapse. Another 3 days later, the HGB was 94 g/L, PLT was 867 × 10^9^/L, CRP was 6.20 mg/L, PCT was 0.05 ng/mL, ESR was 71 mm/h, and ferritin was 97.28 μg/L. The patient showed no symptoms. Another 3 days later, HGB was 94 g/L, PLT was 956 × 10^9^/L, CRP was 3.40 mg/L, PCT was 0.04 ng/ml, and ESR was 61 mm/h. IKD was finally diagnosed, and the basis were list in Table 1 (C). Moreover, this episode might have been a recurrence of IKD, which contributed to the first episode. Aspirin was given at 30 mg/kg/d for 5 days, then continued at 2 mg/kg/d. The HLH treatment was terminated. One month later, aspirin was stopped, when the CRP and ESR returned to normal levels. For 3 months, the patient showed generally good health. She was monitored to check for changes in the PLT level and for the development of CAE.

### The Third Fever Episode

The patient was admitted with a history of rashes for 6 days and fever for 5 days. Skin rashes initially appeared over the trunk, with no obvious causes, and then spread over the entire body. She was referred to a dermatologist and diagnosed with erythema multiforme. Desonide cream and loratadine syrup were prescribed. One day later, she began to develop a fever. The fever spiked four times daily and could be relieved with oral ibuprophen or acetaminophen. The peak axillary temperature was 40.1°C. She displayed no cough, vomiting, or diarrhea. The rashes became more when having a fever. She sometimes complained of pain in the left wrist joint. At an outpatient clinic, the CRP was 25.5 mg/L, and oral cefaclor was prescribed. On the fourth night after fever onset, she was injected with one dose of dexamethasone in the emergency room, due to the high temperature, and the temperature normalized for 1 day before relapsing. Consequently, she was admitted for further evaluation. A physical examination showed that the rashes were scattered all over the body, and some were confluent; the rashes were light red, slightly swollen, without desquamation, and faded upon pressure. The remainder of the physical examination was unremarkable. Laboratory tests revealed that the WBC was 14.71 × 10^9^/L, HGB was 99 g/L, PLT was 329 × 10^9^/L, CRP was 113.3 mg/L, PCT was 0.34 ng/ml, ferritin was >2,000 μg/L, and the ESR was 46 mm/h. Triglycerides, fibrinogen, and sCD25 were normal. The serum was negative for antibodies against CTD or HLA-B27. A blood bacteria culture was performed. Cefmenoxime was administered. Five days later, the fever persisted. The blood culture was negative. A bone aspiration was performed, and the result showed that the marrow was active proliferated, and left moving nucleus in granulocytes, and toxic granulations in cytoplasm of mature granulocytes. SoJIA was diagnosed clinically. Qral methotrexate (5 mg/week), and dexamethasone (2.25 mg, twice daily) were prescribed. The temperature returned to normal in 2 days, and the rashes disappeared in 3 days. She was discharged on the 17th day after admission. CRP, ferritin, and ESR levels gradually declined to normal ranges within 4 months.

We only tested the cytokines IL-1β, IL-6, and interferon-γ (IFN-γ), due to the limited available laboratory tests. The results showed that the cytokines were elevated, even during remissions, and they were much more markedly elevated in the fever phase. The IFN-γ elevation was relatively slight. The data are shown in Table 2.

**Table 2:** Cytokines measured when the fevers occurred.

**Cytokines**	**Reference range**	**At the first time**	**At remission**	**At the second time**	**At remission**	**At the third time**	**At remission**
IL-1β (pg/mL)	0–2	19.1	5.6	13.4	4.6	15.7	3.6
IL-6 (pg/mL)	2.15–12.75	97.54	20.3	80.2	16.82	57.13	14.2
IFN-γ (pg/mL)	2.55–4.37	8.59	4.65	5.58	3.37	6.32	2.15

## Discussion

IKD, HLH, SoJIA, and PFAPA have some overlapping clinical features. Fever is the common symptom, and it has no special pattern. Some signs that accompany the fever might facilitate making a diagnosis after infective fever is excluded. Nonexudative conjunctivitis, mucositis, or desquamation in the hands and feet typically indicate KD, but cervical lymphadenopathy or hepatosplenomegaly is not specific, and might occur in HLH, KD, SoJIA, or PFAPA. Arthritis is an important clue for SoJIA, but it was reported that about 75% SoJIA patients had a delay in onset of arthritis and the history of delay in onset of arthritis ranged from 15 days to over 1 year (the median was 30 days) (19). Not all of these features are necessarily present at the time of diagnosis; consequently, the diagnosis might be delayed in the early phase of the disease before all the classic signs have presented. In particular, high suspicion is necessary for HLH, to ensure early diagnosis and treatment because HLH can be fatal.

Most recurrent fever is caused by infections, but noninfectious diseases should be considered. For IKD, HLH and SoJIA, the fever repeats irregularly. For PFAPA, the fever has a duration of 4–6 days regardless of antibiotics and antipyretics and repeats every 3–5 weeks regularly (20). In China, KD is reported to recur in 1.4% patients (3), and in 2–4% cases in Japan (4). The recurrence usually occurs within 2 years of initial presentation (3, 4). One KD case was reported three times recurrence in 10 years (21). It has reported that over 80% of SoJIA cases to show a recurrent longtime process (5). For our case, FMF, CAPS, TRAPS, and PFAPA was not diagnosed most because of the irregular recurrence of the fevers and relatively severe laboratory changes.

Because the symptoms and signs of these diseases are nonspecific, certain clinical parameters should be investigated carefully. The presence of CAE is a strong indication for KD, but it is also found in HLH that fulfills the criteria for KD (22) or SoJIA (23–27). Age-based anemia, low serum albumin, elevated ALT, and elevated leukocytes are supplemental laboratory criteria for diagnosing IKD in patients with elevated CRP and ESR, with a normal echocardiogram (28, 29). In PFAPA patients, elevated CRP with normal procalcitonin was found (30). However, these criteria are also observed in SoJIA. Yamaguchi criteria for adult Still's disease include some laboratory parameters, and some researchers had discussed the criteria in children and showed the criteria may help to early diagnosis for SoJIA in the “pre-arthritic” phase (19). For the first fever episode, our case fulfilled Yamaguchi criteria [two major criteria (fever and rash) and three minor criteria (Lymphadenoapthy, Liver dysfunction, Negative RF, and ANA test)], regardless of her age. Elevated serum sCD25, which is a criterion for HLH, was also considered a possible indication of subclinical MAS in patients with active SoJIA, when the level was over 7,500 pg/mL (31). When the sCD25 is ≤ 2,400 U/ml, HLH can be excluded, but when the value is >10,000 U/ml, HLH can be diagnosed with a specificity of 93% (32). Elevated CD64 was reported as a potential biomarker for the diagnosis of PFAPA, because elevated CD64 expression was found in PFAPA but not in FMF, CAPS, TRAPS and severe bacterial infections (33). Some genetic deficits contribute to diagnosis are involving the pathogenesis of FMF, CAPS, TRAPS and PFAPA (2, 16). For some parameters, the trend over time can indicate possible early abnormalities, even when the parameter is within the normal range; for example: the blood cell count (34). In our case, the progressive changes in HGB, PLT, fibrinogen, and triglycerides provided a clue to diagnose the first fever episode as HLH.

Recently, serum cytokine and ferritin levels were used as markers to distinguish between KD and SoJIA. Patients with KD typically have a high level of IL-6, a low level of IL-18, and a normal level of serum ferritin; in contrast, patients with SoJIA typically have a low level of IL-6 and high levels of IL-18 and serum ferritin (35–40). And, IL-18 was identified as a useful marker in the differential diagnosis between SoJIA and IKD (36). However, high serum levels of IL-18 and ferritin have also been observed in KD (41). In our case, ferritin was elevated above the detectable limit during the first and third fever episodes, this may indicate that the patient can be a SoJIA from the begin. This finding suggested that ferritin might facilitate the differentiation between HLH, SoJIA, and IKD. IL-6 is likely to facilitate predicting both incomplete and IVIG-nonresponsive KD (42). Moreover, IL-6, IL-10, and IFN-γ were considered to facilitate the early recognition of KD shock syndrome (KSS) in patients with KD. KSS is a severe manifestation of KD, with systolic hypotension or clinical signs of poor perfusion (43). In addition, IL-1, IL-6, and IL-18 cytokines were recognized to play an important role in innate immunity with regard to the pathogenesis of SoJIA (13, 44). Elevated TNF-α, IL-1β, and IL-6 were also observed in PFAPA patients (16). Accordingly, the common involvement of IL-6 might explain the overlapping clinical parameters observed in HLH, IKD, SoJIA and PFAPA. KD had been described as a precursor of secondary HLH, particularly IKD or IVIG-nonresponsive KD (9–11, 45–48), but HLH was also observed before the diagnosis of KD (49). MAS complicated with KD was observed in some cases (50–52). MAS should be taken into account in refractory KD cases (53–55) and in the acute phase of KD (56). There have also been reports of SoJIA with MAS, which was misdiagnosed as KD (26, 27). It has been hypothesized that SoJIA and IKD might belong to a single clinical syndrome with different severities (57). According to our review, the cytokines are commonly elevated in the above diseases and cannot alone confirm a diagnosis today.

In our case, IKD and IVIG–nonresponsive KD were observed before the HLH diagnosis in the first fever episode, but only IKD was observed in the second episode. This observation was consistent with the notion that IVIG-nonresponsive KD was more likely to indicate possible HLH. Although the case manifested like PFAPA when the fever repeated, we did not make the diagnosis because early diagnosis and adequate treatment are more crucial for KD, HLH than PFAPA. Cytokines are not routinely examined in our hospital. The results of tests for IL-1β, IL-6, and IFN-γ showed that IL-6 levels were higher at the onsets of HLH and IKD than at the onset of SoJIA. The IL-6 results from different phases were consistent with previous reports that described different subjects with KD. Elevated IL-1β, IL-6 was observed in the remissions in our case, but it was reported that normal IL-1β, IL-6 level in the remission of PFAPA (16).

## Concluding Remarks

Our case showed a dilemma for differentiating diagnosis for a fever in children. KD and SoJIA are usually considered firstly. And PFAPA is likely to be delayed in diagnosis because of less knowledge and awareness by primary care physicians (58). Surveillance of routine laboratory parameters over time might reveal a trend that indicates possible disease, even when parameter values do not meet diagnostic criteria. Changes in cytokine profiles are promising markers for differentiating recurrent fever diseases in future. An unknown immunological defect for the case may contribute to the recurrent immunological insults, and we are following up the recurrence of fever episode.

## Data Availability Statement

The raw data supporting the conclusions of this article will be made available by the authors, without undue reservation, to any qualified researcher.

## Ethics Statement

Written informed consent was obtained from the patient's parents for publication of this case report.

## Author Contributions

HJ and ZY were responsible for acquisition of the clinical information and writing up of the manuscript. ZY was responsible for writing up of the manuscript.

### Conflict of Interest

The authors declare that the research was conducted in the absence of any commercial or financial relationships that could be construed as a potential conflict of interest.

## Abbreviations

IKD: Incomplete Kawasaki disease
HLH: Hemophagocytic lymphohistiocytosis
SoJIA: systemic-onset juvenile idiopathic arthritis
PFAPA: Periodic Fever, Aphthous Stomatitis, Pharyngitis, and Cervical Adenitis Syndrome
CRP: C-reaction protein
PCT: procalcitonin
ESR: erythrocyte sedimentation rate
ALT: alanine aminotransferase
IVIG: intravenous immunoglobulin
sCD25: soluble CD25
CAE: Coronary artery ectasia
TNF-a: tumor necrosis factor-a
IL-6: interleukin-6.
